# A novel mechanism of regulating breast cancer cell migration via palmitoylation-dependent alterations in the lipid raft affiliation of CD44

**DOI:** 10.1186/bcr3614

**Published:** 2014-02-10

**Authors:** Irina S Babina, Elaine A McSherry, Simona Donatello, Arnold DK Hill, Ann M Hopkins

**Affiliations:** 1 Department of Surgery, Royal College of Surgeons in Ireland, RCSI Education and Research Centre, Beaumont Hospital, Dublin 9, Ireland

## Abstract

**Introduction:**

Most breast cancer-related deaths result from metastasis, a process involving dynamic regulation of tumour cell adhesion and migration. The adhesion protein CD44, a key regulator of cell migration, is enriched in cholesterol-enriched membrane microdomains termed lipid rafts. We recently reported that raft affiliation of CD44 negatively regulates interactions with its migratory binding partner ezrin. Since raft affiliation is regulated by post-translational modifications including palmitoylation, we sought to establish the contribution of CD44 palmitoylation and lipid raft affiliation to cell migration.

**Methods:**

Recovery of CD44 and its binding partners from raft versus non-raft membrane microdomains was profiled in non-migrating and migrating breast cancer cell lines. Site-directed mutagenesis was used to introduce single or double point mutations into both CD44 palmitoylation sites (Cys286 and Cys295), whereupon the implications for lipid raft recovery, phenotype, ezrin co-precipitation and migratory behaviour was assessed. Finally CD44 palmitoylation status and lipid raft affiliation was assessed in primary cultures from a small panel of breast cancer patients.

**Results:**

CD44 raft affiliation was increased during migration of non-invasive breast cell lines, but decreased during migration of highly-invasive breast cells. The latter was paralleled by increased CD44 recovery in non-raft fractions, and exclusive non-raft recovery of its binding partners. Point mutation of CD44 palmitoylation sites reduced CD44 raft affiliation in invasive MDA-MB-231 cells, increased CD44-ezrin co-precipitation and accordingly enhanced cell migration. Expression of palmitoylation-impaired (raft-excluded) CD44 mutants in non-invasive MCF-10a cells was sufficient to reversibly induce the phenotypic appearance of epithelial-to-mesenchymal transition and to increase cell motility. Interestingly, cell migration was associated with temporal reductions in CD44 palmitoylation in wild-type breast cells. Finally, the relevance of these findings is underscored by the fact that levels of palmitoylated CD44 were lower in primary cultures from invasive ductal carcinomas relative to non-tumour tissue, while CD44 co-localisation with a lipid raft marker was less in invasive ductal carcinoma relative to ductal carcinoma *in situ* cultures.

**Conclusion:**

Our results support a novel mechanism whereby CD44 palmitoylation and consequent lipid raft affiliation inversely regulate breast cancer cell migration, and may act as a new therapeutic target in breast cancer metastasis.

## Introduction

Despite improvements in screening and care, breast cancer remains a leading cause of death in women worldwide [1]. Most breast cancer-related deaths arise from tumour metastasis to secondary sites. Cell migration out of the primary tumour is one of the earliest events in the metastatic cascade, and requires coordinated activation of numerous cell adhesion signalling cascades. CD44 is an important cell adhesion molecule with a variety of tissue-dependent functions [2]. CD44 is the major receptor for the extracellular matrix component hyaluronan [3], can act as a co-receptor for growth factors [4] and can organise the actin cytoskeleton through a range of cytoplasmic linker proteins [5]. Because CD44 is involved in a wide spectrum of physiological functions, its dysregulation has been implicated in progression of a variety of cancers [6], including breast cancer. Importantly, CD44 expression has been reported to be elevated in triple-negative mammary tumours and to associate with poor patient outcome [7]. Paradoxically, however, CD44 has been described as a tumour suppressor in some other cancers [8,9]. Some studies attribute this discrepancy to cell-type dependence and differential CD44 subcellular localisation patterns [10,11]. Consequently, in this manuscript we specifically investigate whether regulation of the subcellular localisation of CD44 could account for its regulation of breast cancer cell migration (an early event in the metastatic cascade).

Palmitoylation of two CD44 cysteine residues at positions 286 and 295 in the transmembrane and juxta-membrane regions confers high affinity for cholesterol-enriched and sphingolipid-enriched regions of the cell membrane, termed lipid rafts [11]. Rafts are dynamic membrane regions that cluster together components of many signalling cascades known to be altered in cancer [12,13]. The CD44 cytoplasmic tail helps organise the actin cytoskeleton via cytoplasmic actin-binding linker proteins, including members of the ezrin/radixin/moesin family, merlin, annexin II and ankyrin. The intrinsic role of actin reorganisation in cellular adhesion and migration underlies why dysregulation of CD44-based signalling has been associated with the pathophysiological manifestations of cancer dissemination and metastasis [14,15]. However, the specific contribution of lipid rafts to the regulation of CD44-dependent adhesion/migration signalling remains incompletely understood. Several reports have linked CD44 lipid raft affiliation to cell survival and oncogenic signalling. CD44–hyaluronan interactions have been suggested to take place in the lipid rafts of breast cancer cells to facilitate oncogenic signalling [16], while CD44 interactions with the cytoplasmic binding partner merlin have been shown to inhibit cancer cell growth [17].

Having recently shown that CD44 affiliation with lipid rafts is reduced in migrating breast cancer cells and hypothesised that translocation outside rafts permits cell migration [18] we set out to examine whether dynamic alterations in CD44 palmitoylation could directly drive cell migratory events by modifying CD44 raft affiliation. We show for the first time that manipulation of CD44 raft affiliation via site-directed mutagenesis of palmitoylation sites influences the migration of invasive breast cancer cells, and is sufficient to induce a motile phenotype and functions in non-invasive cells. Furthermore, we demonstrate temporal reductions in palmitoylated CD44 during stimulated migration of breast cancer cells. Importantly, we provide evidence that reductions in CD44 palmitoylation are paralleled by increased CD44 co-association with its binding partner ezrin. Our findings in cell lines are supported by data from breast primary cell cultures, in which lower palmitoylation and less co-localisation of CD44 with raft markers correlates with more aggressive cancers. Our data are consistent with a novel model whereby palmitoylation-induced retention of CD44 within lipid rafts exerts a suppressive effect on breast cancer cell migration. Post-translational events of this nature offer a novel (non-genomic) possibility to help explain the controversy that CD44 expression levels alone do not always correlate directly with prognosis in different cancers. In conclusion, therefore, we speculate that pharmacological targeting of CD44 palmitoylation may offer a fresh strategy to reduce cancer cell dissemination during the early stages of metastasis.

## Methods

### Cell culture and transfection

Human breast cancer cell lines MCF-10a and MDA-MB-231 were obtained from ATCC (LGC Standards, Teddington UK). MDA-MB-231 cells were cultured in Dulbecco’s modified Eagle’s medium (Sigma-Aldrich, Arklow, Ireland) supplemented with 10% foetal bovine serum, 2 mM l-glutamine, 100 U/ml penicillin and 100 μg/ml streptomycin. MCF-10a cells were cultured in Dulbecco’s modified Eagle’s medium/F12 Ham (Sigma-Aldrich) supplemented with 100 U/ml penicillin, 100 μg/ml streptomycin, 5% horse serum, 0.5 μg/ml hydrocortisone, 0.1 μg/ml cholera toxin, 10 μg/ml insulin and 20 ng/ml epidermal growth factor (all Sigma-Aldrich). Mammary tumour primary cell cultures were generated as described elsewhere [19] from mastectomies or lumpectomies obtained with full consent and prior ethical approval (Beaumont Hospital Medical Ethics (Research) Committee) from symptomatic patients undergoing breast cancer surgery in Beaumont Hospital. Primary cultures were grown in mammary epithelial growth medium (Lonza, Verviers, Belgium), prepared according to the manufacturer’s guidelines. For transient transfections (48 hours, unless indicated otherwise) of plasmid DNA into the cells, jetPRIME transfection reagent (Source Bioscience, Dublin, Ireland) was used according to the manufacturer’s guidelines. Untransfected control cells were incubated with DNA-diluting buffer alone. Transfected cells were selected using chloramphenicol (Sigma-Aldrich) dissolved directly in culture medium, at 300 μg/ml for MDA-MB-231 and at 500 μg/ml for MCF-10a, as determined by kill curve proliferation assays (data not shown). Cells transfected with wild-type CD44 acted as a conditional control. All cells were cultured in a humidified incubator at 37°C and 5% carbon dioxide, and were confirmed to be mycoplasma free by quarterly testing.

### Antibodies and reagents

Mouse anti-human CD44 primary antibody, detecting all isoforms of the protein, was obtained from R&D Systems (Abingdon, UK) and used for western blot analysis. For immunofluorescence and immunoprecipitation, mouse CD44 antibody was purchased from Santa Cruz Biotechnology (Heidelberg, Germany). Antibodies to detect flotillin-1 and ezrin (mouse) were from BD Biosciences (Oxford, UK). Mouse transferrin receptor antibody and goat anti-rabbit horseradish peroxidase (HRP) were from Cell Signaling Technologies (Danvers, MA, USA). Antibodies against radixin, moesin, annexin II and merlin (rabbit) were from GeneTex, Inc. (Irvine, CA, USA). Rabbit flotillin-1 antibody (immunofluorescence), anti-actin primary antibody and goat anti-mouse HRP secondary antibody were from Sigma-Aldrich. Streptavidin HRP was from ThermoScientific (Ballycoolin, Ireland) and Millipore (Cork, Ireland).

### Plasmid constructs

A pOTB7 plasmid encoding wild-type human CD44 (imaGenes; Source Bioscience) was grown on Luria-Bertani agar plates with 20 μg/ml chloramphenicol and subsequently in Luria-Bertani broth with the same antibiotic concentration. Plasmid extraction was carried out using Qiagen Midi-Prep kits following the manufacturer’s protocols (Qiagen, Manchester, UK). This preparation was used as template genetic material for site-directed mutagenesis of CD44 palmitoylation sites. Single point mutations were obtained using the QuikChange Site-Directed Mutagenesis kit (Agilent Technologies Ireland Ltd, Cork, Ireland). Cys286 was mutated to either serine (C286S) or alanine (C286A). A double mutation was achieved using the mutated Cys286 DNA as a template for introducing a mutation at Cys295 to alanine. The mutagenesis primers were obtained from Eurofins MWG Operon (Ebersberg, Germany) and contained the sequences presented in Table 1. All mutations were confirmed by sequencing of the synthesised plasmids (Source Bioscience) and alignment using BLAST software (National Institute of Health, Bethesda, MD, USA). It is noteworthy that mammalian expression of pOTB7 plasmid inserts is normally achieved by subcloning via the Gateway expression system (Life Technologies, Paisley, UK). However, based on the precedent that mammalian expression can be successfully achieved without the subcloning step [20] (albeit via an unknown mechanism), we did not use the Gateway step.

**Table 1:** Oligonucleotide primers used for site-directed mutagenesis

**Name**	**Oligonucleotide (5′ to 3′)**
C286A Forward	GCT TTG ATT CTT GCA GTT GCC ATT GCA GTC AAC AGT CG
C286A Reverse	CGA CTG TTG ACT GCA ATG GCA ACT GCA AGA ATC AAA GC
C286S Forward	GCT TTG ATT CTT GCA GTT TCC ATT GCA GTC AAC AGT CG
C286S Reverse	CGA CTG TTG ACT GCA ATG GAA ACT GCA AGA ATC AAA GC
C295A Forward	CAG TCG AAG AAG GGC TGG GCA GAA GAA AAA GC
C295A Reverse	GCT TTT TCT TCT GCC CAG CCC TTC TTC GAC TG

Successful expression of the CD44 mutants in MDA-MB-231 transfectants was confirmed by sequencing of polymerase chain reaction products of reverse-transcribed RNA using the following primers (5′ to 3′, codon underlined): C286A mutant (mutation of T-G at position 856), forward GCAGATCGATTTGAATATAACCTGC and reverse CTGTTGACTGCAAT*GCC*AACTG; and SA mutant containing C286S and C295A mutations (mutation of G-C at position 857 and TG-GC at positions 883 and 884), forward GCAGATCGATTTGAATATAACCTGC and reverse GCTTTTTCTTCTGCCC*AGC*C. Polymerase chain reaction product and sequencing results are shown in Figure S1 in Additional file 1.

### Triton X-100 insolubility assay

Cells were grown to confluence in six-well plates. Detergent-soluble fractions (enriched in nonraft cellular components) were obtained following 30 minutes of incubation at 4°C in lysis buffer (100 mM KCl, 3 mM NaCl, 3.5 mM MgCl_2_, 10 mM HEPES) containing 1% Triton X-100 and protease and phosphatase inhibitor cocktails (Sigma-Aldrich). The detergent-insoluble pool (enriched in lipid rafts) was subsequently collected via scraping the wells in one-half volume of lysis buffer. After determination of protein concentrations using Bicinchoninic acid assay, fractions of equivalent protein concentration were analysed by immunoblotting.

### Lipid raft extraction by discontinuous sucrose gradient fractionation

All steps were carried out at 4°C. Cells were lysed in calcium-positive and magnesium-positive Hank’s balanced salts solution (Sigma-Aldrich) supplemented with 1% Triton X-100 (Roche Diagnostics, West Sussex, UK) and a protease inhibitor cocktail (Sigma-Aldrich). Lysates were dounced ×20 and triturated ×20 using a 26-gauge needle, and were then mixed in a 1:1 ratio with 90% (w/v) sucrose (dissolved in Hank’s solution). Then 4 ml was loaded into an ultracentrifuge tube (Roche Diagnostics) and sequentially overlain with equal volumes of 30%, 20% and 5% (w/v) sucrose. Preparations were ultracentrifuged in a Beckman Optima L-100 K ultracentrifuge using an SW41Ti rotor (~260,000 × *g*/19 hours/4°C). One-millilitre fractions were collected from the top and analysed as described previously [21]. Briefly, sucrose density was estimated using a refractometer. Alkaline phosphatase activity, to identify lipid raft-enriched fractions, was quantitated by incubating 1:10 volumes of fraction:*p*-nitrophenyl phosphate substrate (Sigma-Aldrich) for 30 minutes, and measuring absorbance at 405 nm. Additionally, each fraction was tested for expression of lipid raft and nonraft markers Flotillin-1 and transferrin receptor (TfR), respectively, using immuno-dot blot.

### Immunoblotting

Equal protein concentrations were loaded onto 10% Tris–HCl gels and subjected to SDS-PAGE. Protein was transferred onto a nitrocellulose membrane by wet transfer at 100 V/1 hour, incubated in blocking buffer (5% skim milk or bovine serum albumin in TBS–0.1% Tween-20) for 1 hour and incubated with primary antibody overnight at 4°C. For dot blots, 2 μl each fraction was loaded directly onto membranes, blocked, and incubated with primary antibody overnight. After washing, membranes were incubated with an appropriate HRP-conjugated secondary antibody for 1 hour at room temperature. Membranes were developed by exposure to X-ray film (Sigma-Aldrich) using enhanced chemiluminescent reagents (PerkinElmer Life Sciences, Waltham, MA, USA). The same membranes were probed for expression of other proteins via stripping primary antibody complexes with 0.7% 2-β-mercaptoethanol (Sigma-Aldrich) as described previously [21].

### Acyl-biotin exchange (1-biotinamido-4-(4′-(maleimidomethyl)cyclohexanecarboxamido)butane assay)

This method was adapted from a previous publication [22]. Briefly, CD44 protein was immunoprecipitated from lysates via overnight incubation at 4°C with 3 μg mouse anti-human CD44 antibody as described elsewhere [18]. Antibody–protein complexes were collected by 3-hour rotation at 4°C with 50 μg protein G-sepharose (Sigma-Aldrich), in the presence of 50 mM *N*-ethylmaleimide (Sigma-Aldrich), to covalently block sulfhydryl groups. Complexes were subsequently divided into two fractions, for treatment with and without 1 M hydroxylamine (Sigma-Aldrich) at room temperature for 1 hour (pH 7.40) to cleave free palmitate groups. Samples were then incubated with 1 μM EZ-link biotin-1-biotinamido-4-(4′-(maleimidomethyl)cyclohexanecarboxamido)butane (biotin-BMCC; ThermoScientific) at pH 6.2 for 1 hour at room temperature, to label the reactive cysteine residues. Labelled CD44 was released from the beads via incubation (100°C/5 minutes) in 2× reducing Lamelli sample buffer, and was subjected to SDS-PAGE and immunoblotting. Biotin-BMCC-labelled CD44 was detected with streptavidin-HRP, followed by antibody, to visualise total CD44.

### Scratch-wound migration assay

Confluent cells were subjected to ligand-independent scratch-wound assays as described previously [18]. Briefly, cells were wounded by a single scratch using a sterile p200 pipette tip attached to suction, rinsed with phosphate-buffered saline and allowed to migrate in serum-free medium in a humidified incubator at 37°C/5% carbon dioxide. Wells were imaged immediately after wounding (time = 0) and the precise location subsequently photographed every 2 hours for 6 to 8 hours. Wound widths were measured using Scion Image software (Scion Corporation Ltd, Frederick, MD, USA) at every time point, and the percentage wound closure was calculated by expressing wound widths relative to the cognate time = 0 measurement for each condition. Migration versus time graphs were subsequently plotted using SigmaPlot (Systat Software Inc, London, UK).

### Image analysis and statistical analysis

Densitometric analysis was performed on western blot films (exposed for equivalent times) using ImageJ software (National Institute of Health, Bethesda, MD, USA). Raw values were used for statistical analysis from a minimum of three independent experiments, and averages are expressed as the percentage of internal control (nonmigrating condition for untransfected cells, and CD44WT for transfected cells). *P* values were calculated using equal variance two-tailed Student’s *t* tests. For migration assays, two-way analysis of variance tests were performed across all time points using GraphPad Prism (GraphPad Software, La Jolla, CA, USA). Results were considered significant when *P < 0.05.*

## Results

### CD44 raft affiliation varies across differentially-invasive breast cancer cells

**Figure 1:**
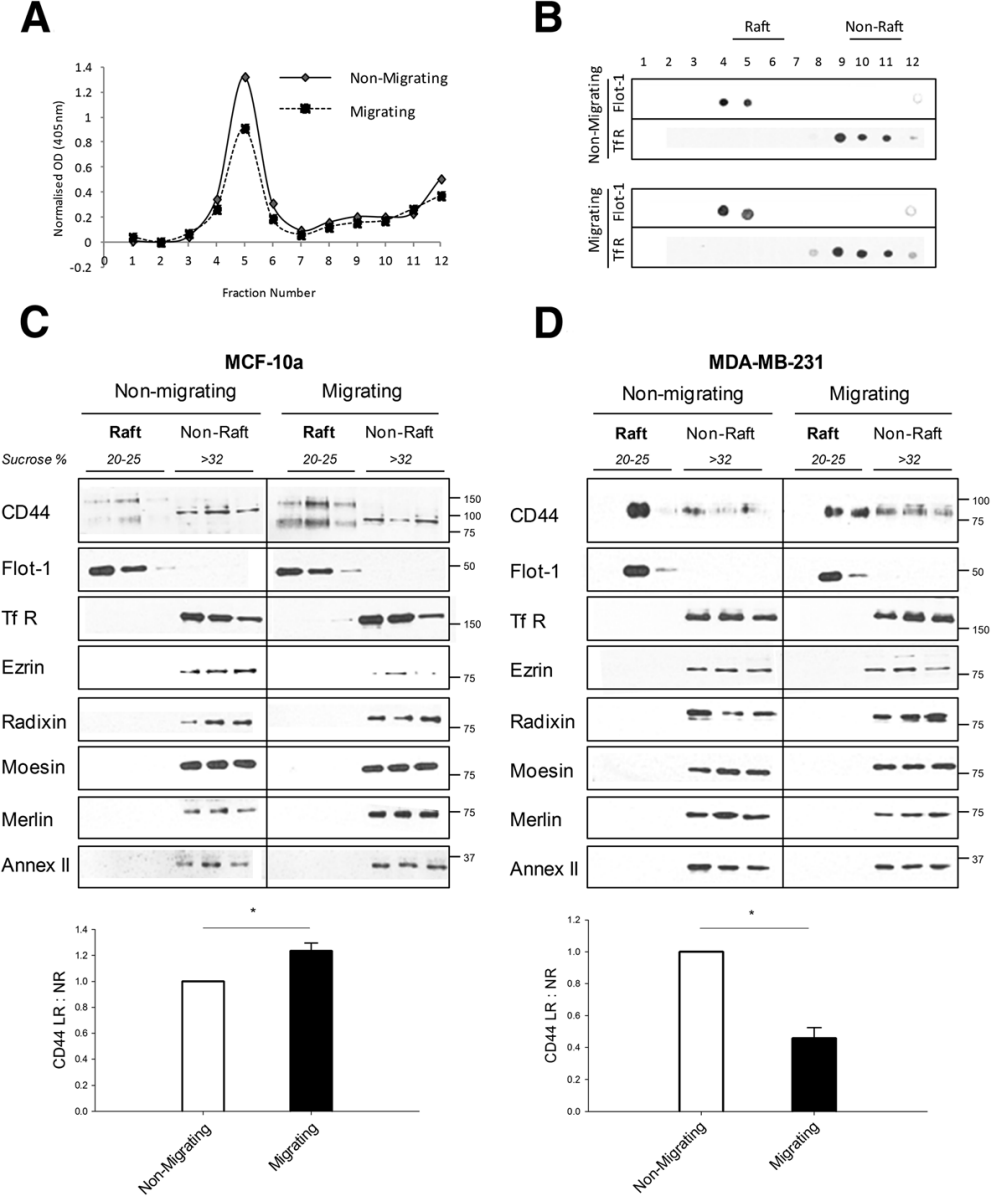
**CD44 affiliation with lipid rafts is reduced during migration of highly-invasive breast cancer cells.** Sucrose density gradient fractionation was used to isolate lipid rafts from nonmigrating (a confluent cell monolayer) versus migrating (2 hours post scratch-wounding) MDA-MB-231 cells. **(A)** Raft fractions were identified on the basis of peak biochemical activity of the lipid raft-affiliated enzyme alkaline phosphatase. **(B)** Enrichment of the marker proteins Flotillin-1 (Flot-1) and transferrin receptor (TfR) by immuno-dot blot was further used to define the identity of respectively raft versus nonraft fractions. **(C)** The western blot expression profile of CD44 and its binding partners in MCF-10a normal-like breast cells revealed increased recovery of raft-affiliated CD44 after 2 hours of migration compared with nonmigrating controls. This was verified by calculation of the raft affiliation ratio from the quantification of three independent experiments (histogram). The CD44 binding partners tested were exclusively recovered from nonraft fractions. **(D)** CD44 recovery from lipid raft fractions of highly-invasive MDA-MB-231 breast cancer cells was reduced in migrating relative to nonmigrating conditions; and was paralleled by increased CD44 recovery from nonraft fractions in migrating conditions. This observation was verified by calculation of the raft affiliation ratio from the quantification of three independent experiments (histogram). The CD44 binding partners tested were exclusively recovered from nonraft fractions. Error bars, standard error of the mean; n = 3 experiments. *P < 0.05, Student’s t test. NR, nonlipid raft; OD, optical density.

We previously reported a decrease in CD44 lipid raft affiliation in migrating MDA-MB-231 and Hs578T cell lines [18]. In the present study we firstly compared the raft affiliation status of CD44 in the non-invasive, normal-like cell line MCF-10a and the highly-invasive cell line MDA-MB-231. Isopycnic sucrose density gradient fractionation was used to isolate rafts under migrating versus nonmigrating conditions, and fractions were analysed for activity of the raft-affiliated enzyme alkaline phosphatase. The enzyme was most active in fractions 4 to 6 (Figure 1A), suggesting enrichment of rafts at sucrose densities of 20 to 25%. Immuno-dot blots (Figure 1B) confirmed partitioning of the raft marker protein Flotillin-1 to the same fractions, and exclusive detection of the nonraft marker TfR at higher sucrose densities (Fractions 8 to 12; >30% sucrose). Thereafter, we refer to fractions 4 to 6 as lipid raft fractions and to fractions 9 to 11 as nonraft fractions.

Western blotting was used to compare the affiliation of CD44 and its binding partners with rafts under nonmigrating and migrating conditions. In MCF-10a cells, CD44 recovery in raft fractions was increased in migrating versus nonmigrating cells (Figure 1C). A high molecular weight CD44 band was also noted in raft fractions. Integrated band densities from three independent experiments were used to calculate the ratio of CD44 affiliation with lipid rafts (described previously [18]). This value (CD44 lipid raft:nonlipid raft) is CD44 normalised to Flotillin-1 as a loading control for raft fractions, divided by CD44 normalised to TfR as a loading control for nonraft fractions. An increase in the ratio reflects increased CD44 raft affiliation. In this study we concentrated on the standard isoform of CD44, and this isoform alone was used for estimation of the CD44 lipid raft:nonlipid raft ratio. A significant increase in the ratio was noted in migrating MCF-10a cells (Figure 1C, lower panel; *P < 0.05*). The CD44 binding partners ezrin, radixin, moesin, merlin and annexin II were detected only in nonraft fractions (Figure 1C). In contrast to MCF-10a cells, CD44 recovery in nonraft fractions of MDA-MB-231 cells was increased after the induction of migration (Figure 1D). This was accordingly quantified as a significant decrease in the CD44 raft affiliation ratio (Figure 1D, lower panel; *P < 0.05*). Furthermore, using isogenic cell line Hs578T and its more invasive derivative Hs578Ts(i)_8_[23], we observed a relatively higher proportion of CD44 outside lipid rafts in the Hs578Ts(i)_8_ cells (data not shown). As with MCF-10a, all of the CD44 binding partners tested were recovered exclusively from nonraft fractions in nonmigrating and migrating cells.

### CD44 palmitoylation-impaired mutants have reduced raft affiliation

**Figure 2:**
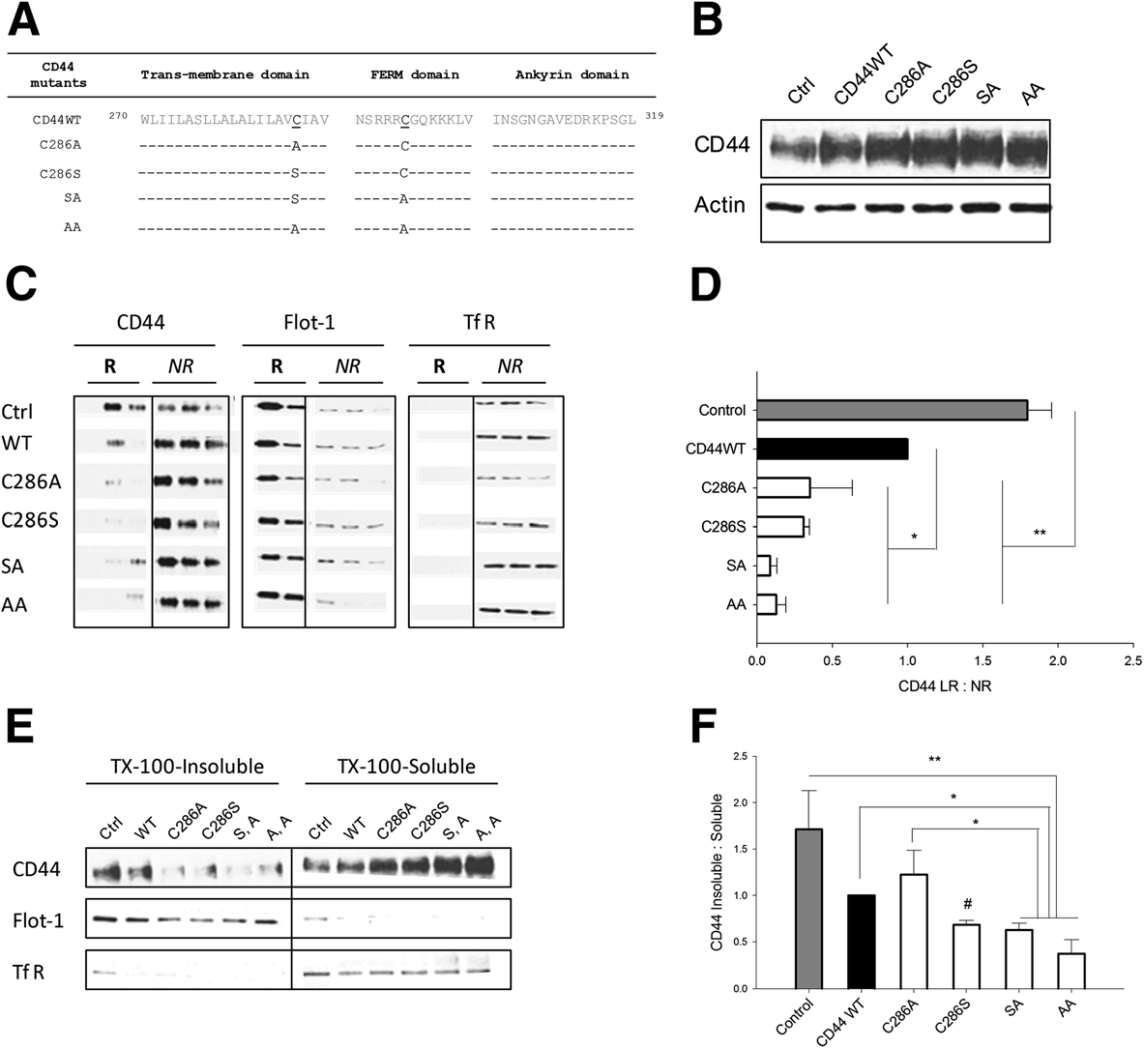
**CD44 palmitoylation-impaired constructs have reduced affiliation with lipid rafts. (A)** Schematic representation of palmitoylation-impaired CD44 constructs generated by site-directed mutagenesis of human CD44. Single-site mutants were termed C286A and C286S, while double-site mutants (C286S + C295A, C286A + C295A) were termed SA and AA respectively. **(B)** MDA-MB-231 cells were transfected with full-length CD44 (CD44WT) or CD44 single-site (C286A, C286S) and double-site (SA, AA) palmitoylation-impaired mutants and were selected for 48 hours. Whole cell lysates revealed increased expression of CD44 in the transfected cells. **(C)** Full-scale lipid raft extractions confirmed reductions in raft-affiliated CD44 in cells expressing the mutant constructs relative to controls. **(D)** Calculation of the lipid raft:nonlipid raft (LR:NR) ratio for multiple experiments confirmed statistically significant reductions in CD44 raft affiliation upon expression of the CD44 palmitoylation-impaired mutants. **(E)** Triton X-100-insoluble and Triton X-100-soluble fractions were isolated and confirmed to be enriched in respectively lipid raft (Flotillin-1 (Flot-1)) or nonraft (transferrin receptor (TfR)) markers. CD44 recovery from raft-enriched fractions was reduced in cells overexpressing mutant CD44, and paralleled by increased recovery of CD44 in nonraft fractions. **(F)** Calculation of the affiliation ratio of CD44 with detergent-insoluble versus detergent-soluble fractions confirmed reductions in raft-affiliated CD44 from cells expressing mutant constructs relative to controls. *Error bars, standard error of the mean; n = 3.*^*#*^P < 0.05 (C286S vs. control); *P < 0.05; **P < 0.01, Student’s t test. HAM, hydroxylamine; WT, wild-type.

Having observed reduced CD44 raft affiliation in migrating highly invasive cells, we sought to directly manipulate CD44 raft affiliation to test its impact on cell motility. Site-directed mutagenesis was used to introduce point mutations into one (C286A or C286S) or both (C286A,295A (AA) and C286S,295A (SA)) cysteine residues that control CD44 palmitoylation and incorporation into rafts (Figure 2A). MDA-MB-231 cells were transiently transfected with palmitoylation-impaired CD44 constructs, negatively selected for 48 hours and subjected to sucrose density gradient fractionation. Whole cell lysate analysis by western blotting revealed increased expression of CD44 in transfected cells (Figure 2B), although we were not able to distinguish between endogenous and exogenous protein. Raft and nonraft fractions were identified by enrichment in respectively Flotillin-1 and TfR (Figure 2C). Cells expressing palmitoylation-impaired mutants had less CD44 in lipid raft fractions and correspondingly more recovered from nonraft domains. Compared with either CD44WT-expressing or control cells, the CD44 raft affiliation ratio was significantly reduced in cells expressing single or double palmitoylation mutants (Figure 2D, *P < 0.05*). Given the requirement for large quantities of material for raft extraction preparations, Triton X-100 insolubility assays were investigated as a surrogate to estimate relative amounts of CD44 in detergent-insoluble (putatively raft-enriched) and detergent-soluble (putatively nonraft-enriched) pools. As expected, Triton X-100-insoluble fractions were enriched in Flotillin-1, while detergent-soluble fractions were enriched in TfR (Figure 2E). MDA-MB-231 cells expressing wild-type CD44 had a similar CD44 solubility/insolubility profile compared with control cells. In contrast, cells transfected with CD44 palmitoylation mutants showed decreased CD44 recovery in detergent-insoluble pools and a corresponding increase in recovery from detergent-soluble pools. Calculation of the affiliation ratio of CD44 with detergent-insoluble fractions (‘CD44 Insoluble:Soluble’, Figure 2F) revealed reductions in cells expressing single or double palmitoylation mutants versus control or CD44WT-overexpressing cells (*P < 0.05* for all). Since these findings mirrored results from sucrose gradient raft extractions, the simpler detergent-extraction approach was subsequently used.

### Direct reduction of CD44 palmitoylation promotes a migratory phenotype

**Figure 3:**
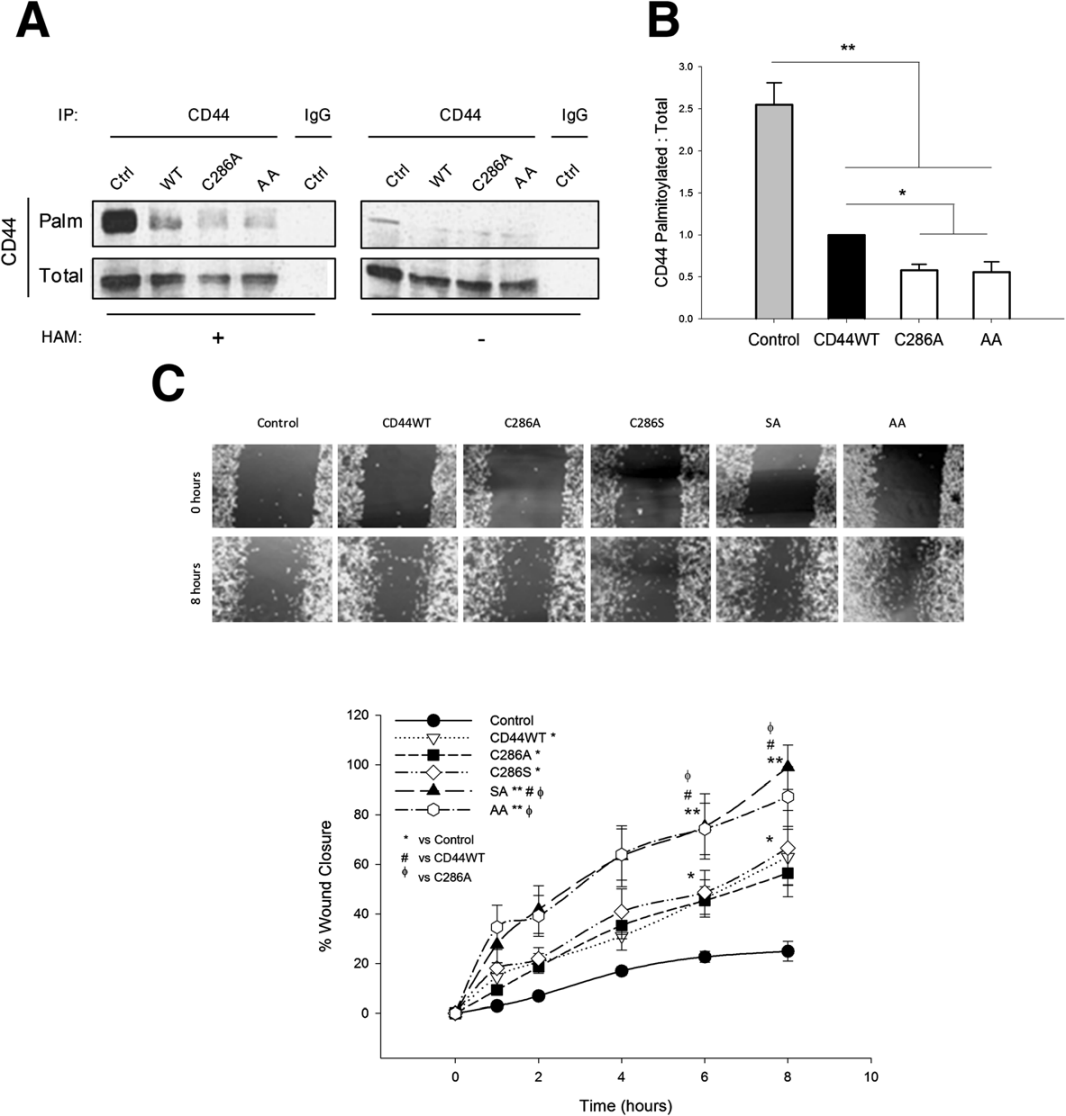
**Reduced CD44 palmitoylation is paralleled by increased cell migration. (A)** Biotin-1-biotinamido-4-(4′-(maleimidomethyl)cyclohexanecarboxamido)butane (biotin-BMCC) assays were used to measure palmitoylated CD44 (CD44-Palm) in cells expressing representative single and double palmitoylation mutants, and were compared with total CD44 levels (CD44-Total). Isotype-matched IgG was used as a negative control for CD44 immunoprecipitations (IPs), and omission of hydroxylamine (HAM) reagent was a BMCC-negative control. CD44-Palm was reduced in cells overexpressing CD44 palmitoylation-impaired mutant constructs. **(B)** Densitometric quantification of multiple experiments confirmed significant reductions in CD44-Palm relative to CD44-Total in mutant cells. Error bars, standard error of the mean (SEM); n = 3. *P < 0.05; **P < 0.01, Student’s t test. **(C)** Phase contrast micrographs of scratch-wound migration assays performed in MDA-MB-231 cells transfected for 48 hours with CD44WT or CD44 single-site (C286A, C286S) or double-site (SA, AA) palmitoylation-impaired mutants. The graph was constructed by expressing wound width measurements at each time point relative to its cognate time = 0 value. Cell migration was significantly enhanced in mutant-expressing cells compared with control cells, as indicated in the graphical representation of multiple experiments. *Error bars, SEM; n = 3. #*φ*P < 0.05; **P < 0.01, two-way analysis of variance. WT, wild-type.

To confirm that changes in CD44 raft distribution reflected reductions in its palmitoylation, biotin-BMCC assays were performed on nonmigrating cells overexpressing representative single and double palmitoylation mutants (Figure 3A,B). Free palmitate groups were cleaved from CD44 immunoprecipitates using hydroxylamine, whereupon reactive cysteines were labelled with biotin-BMCC and detected with streptavidin. Biotin-labelled (palmitoylated) CD44 was lower in cells expressing palmitoylation mutants versus wild-type or control cells. Band quantitation of palmitoylated versus total CD44 confirmed significant reductions in palmitoylated CD44 in MDA-MB-231 cells overexpressing single and double mutants (Figure 3B). To determine whether forced exclusion of CD44 from rafts directly promoted cell migration, scratch-wound assays were performed in MDA-MB-231 cells transiently transfected with CD44 palmitoylation-impaired mutants (Figure 3C). Overexpression of CD44WT or CD44 single-site palmitoylation mutants doubled cell migration compared with controls. Overexpression of CD44 double-site palmitoylation mutants improved cell migration fourfold relative to control cells (Figure 3C) or twofold relative to CD44WT or single mutant cells (*P < 0.05*).

**Figure 4:**
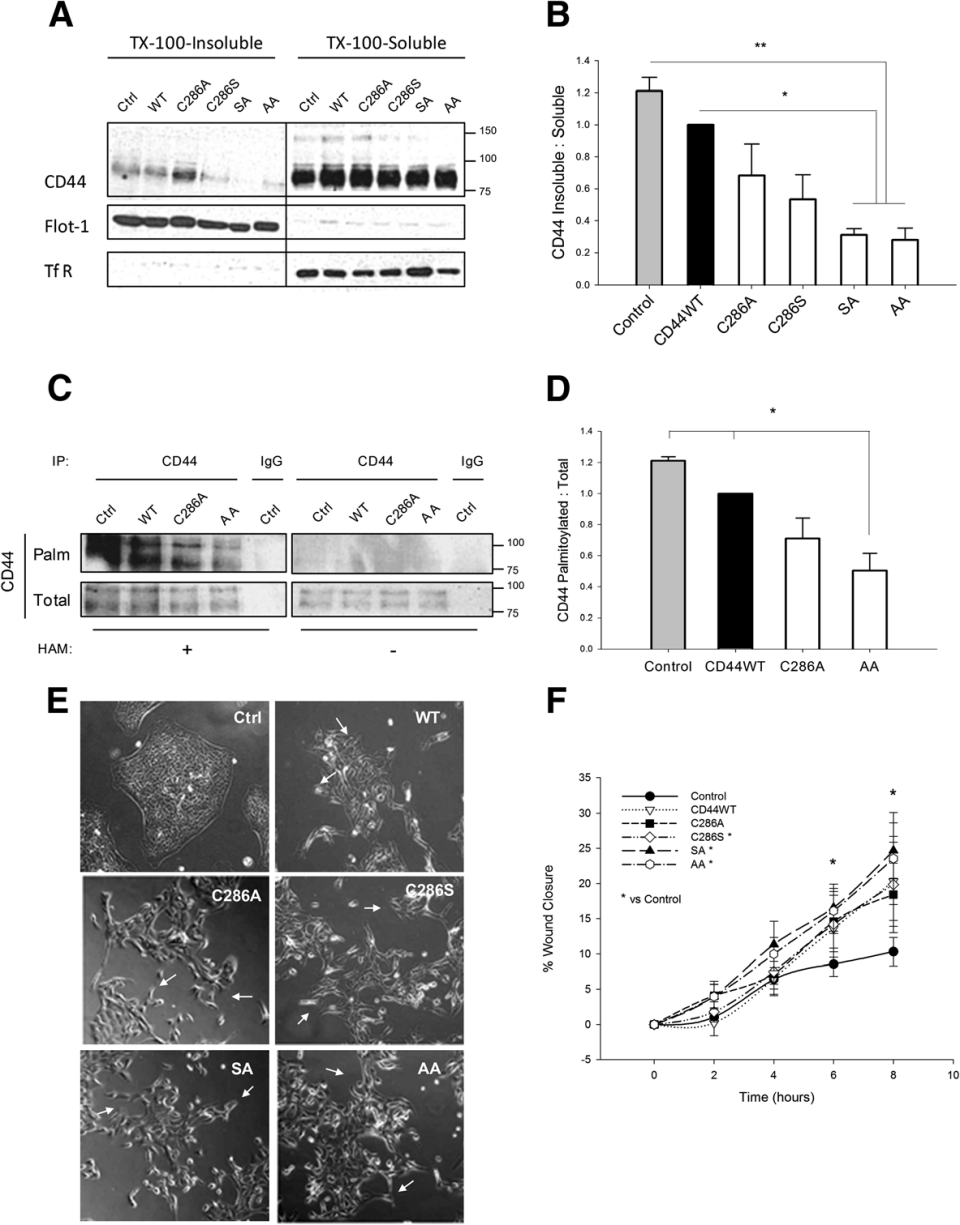
**Reducing CD44 is sufficient to alter normal phenotype in breast cells.** MCF-10a cells were transfected for 48 hours with full-length CD44 (CD44WT), and single-site (C286A, C286S) or double-site (SA, AA) palmitoylation-impaired mutants. **(A)** Triton X-100-insoluble and Triton X-100-soluble fractions were isolated, and were confirmed to be enriched in respectively lipid raft (Flotillin-1 (Flot-1)) and nonraft (transferrin receptor (TfR)) marker proteins. Overexpression of mutant CD44 reduced CD44 recovery from raft-containing fractions compared with that in untransfected controls and CD44WT-expressing cells. **(B)** Calculated ratios of CD44 affiliation with detergent-insoluble fractions reflected significant reductions in CD44 raft affiliation in mutant cells compared with untransfected control cells, and furthermore in CD44 double-site mutants compared with CD44WT. **(C)** CD44 palmitoylation was assessed by 1-biotinamido-4-(4′-(maleimidomethyl)cyclohexanecarboxamido)butane (BMCC) assay in CD44 immunoprecipitates of control, CD44WT-expressing and CD44 mutant-expressing cells. Palmitoylated CD44 was detected with streptavidin (CD44-Palm), and total CD44 detected using CD44 antibody (CD44-Total). No CD44 was immunoprecipitated in the isotype-matched IgG control lanes, and no palmitoylated CD44 was detected in the hydroxylamine (HAM)-negative control conditions. In the HAM-positive condition, palmitoylated CD44 was reduced in mutant-expressing cells. **(D)** Quantification of palmitoylated CD44 (as a ratio of total CD44) revealed significant reductions in cells overexpressing a double-site CD44 palmitoylation-impaired mutant (AA) relative to control or CD44WT-expressing cells (*P < 0.05; **P < 0.01, Student’s t test). **(E)** Phase-contrast micrographs 48 hours post transfection revealed a change in the morphology of control MCF-10a colonies (Ctrl) following CD44 overexpression, with a protrusive, disseminated epithelial-to-mesenchymal transition-like appearance in all of the CD44-overexpressing cells (arrows), which was particularly pronounced in those expressing the CD44 palmitoylation-impaired mutants. **(F)** Migration was measured via scratch-wound assays, in which wound widths at each time point were expressed relative to their cognate measurement at time = 0 for each condition. These assays revealed significant enhancements in the migratory capacity of MCF-10a cells expressing CD44 palmitoylation-impaired mutants relative to control cells (*P < 0.05, two-way analysis of variance). Error bars, standard error of the mean; n = 3 experiments. WT, wild-type.

We next tested whether forced exclusion of CD44 from rafts was sufficient to induce a migratory phenotype in non-invasive breast cells. CD44 palmitoylation-impaired mutants were overexpressed in histologically normal MCF-10a cells, whereupon Triton X-100 insolubility assays confirmed reduced partitioning to raft fractions (Figure 4A). CD44 recovery in detergent-insoluble pools was virtually abolished in cells expressing double-site mutants (Figure 4B, *P < 0.05* vs. CD44WT and *P < 0.01* vs. control). Biotin-BMCC assays confirmed reductions in palmitoylated CD44 in cells overexpressing CD44 palmitoylation-impaired mutants (Figure 4C,D). Relative to control MCF-10a cells, overexpression of either CD44WT or CD44 palmitoylation-impaired mutants disrupted characteristically tight colonies in these cells, inducing cell scattering in a manner reminiscent of that reported to occur in epithelial-to-mesenchymal transition (EMT; Figure 4E) in conjunction with reduced expression of the epithelial marker EpCAM and enhanced expression of the mesenchymal marker vimentin (Figure S2 in Additional file 2). This was accompanied by increased cell migration, particularly in cells overexpressing CD44 double-site mutants (Figure 4F, *P < 0.05* vs. control). Mutant deselection after 48 hours followed by two subcultures in normal medium normalised both the CD44 raft affiliation ratio (Figure S3A,B,C,D in Additional file 3) and cell migration (Figure S3E,F in Additional file 3) to control levels.

### Reduced CD44 palmitoylation facilitates ezrin binding and correlates with invasive phenotype

**Figure 5:**
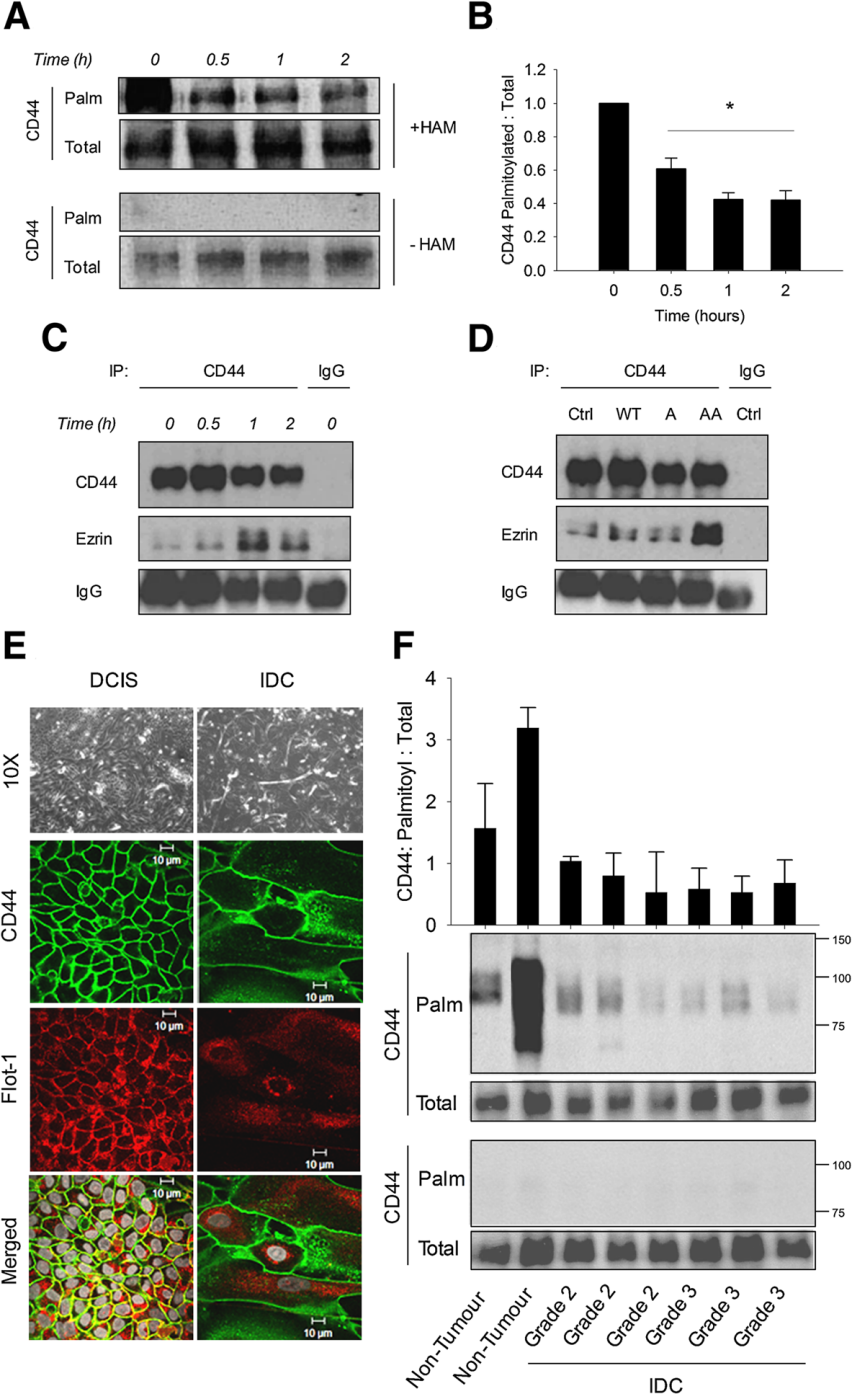
**Decreased CD44 palmitoylation is paralleled by increased co-association with ezrin. (A)** CD44 immunoprecipitates from different MDA-MB-231 migration time points were probed for palmitoylated CD44 by 1-biotinamido-4-(4′-(maleimidomethyl)cyclohexanecarboxamido)butane (BMCC) assay. Palmitoylated CD44 was detected using streptavidin (CD44-Palm), and total CD44 detected using a CD44 primary antibody (CD44-Total). The absence of hydroxylamine (HAM) treatment was a negative control for palmitoylated CD44. CD44-Palm levels decreased over the migration time course. **(B)** Quantification of CD44-Palm as a ratio of CD44-Total revealed significant time-dependent reductions during migration. Error bars, standard error of the mean; n = 3 experiments. *P < 0.05, Student’s t test.**(C)** CD44 was immunoprecipitated from whole cell lysates of MDA-MB-231 cells at the indicated migration time points, with IgG as an internal control. Immunoprecipitates were probed for CD44 and ezrin, with the IgG heavy chain band intensity considered as a loading control. Ezrin-CD44 co-immunoprecipitation (IP) was increased after 1 and 2 hours of cell migration compared with nonmigrating cells (time = 0). **(D)** CD44 was immunoprecipitated from migrating (2 hours) untransfected MDA-MB-231 (Ctrl), WT-expressing, single and double palmitoylation mutant-expressing cells. The latter cells had a notable increase in CD44-ezrin co-association. IP experiments are representative of three independent experiments. **(E)** Confluent breast primary cells were stained for CD44 (green) and Flotillin-1 (Flot-1, red). Nuclei were stained with DAPI and are shown in grey. Ductal carcinoma *in situ* (DCIS) primary cells demonstrated the highest co-localisation of the two proteins compared with invasive ductal carcinomas (IDC). **(F)** Whole cell lysates of primary cultures from two nontumour (NT), and six IDC of tumour grade 2 and 3 (three of each) were subjected to BMCC assays to compare palmitoylated CD44 levels. Most palmitoylated CD44 (CD44-Palm) was recovered from NT cultures, while all patient IDC cultures displayed comparatively little CD44-Palm. Palmitoylated CD44 levels densitometrically normalised to total CD44 (CD44-Total) further demonstrated increased levels of CD44-Palm in NT samples*. Error bars, standard deviation of duplicates in one experiment*.

Since modulation of CD44 palmitoylation reversibly modified cell migration, we questioned the reverse – whether stimulation of cell migration might reduce CD44 palmitoylation to facilitate its extra-raft translocation. CD44 palmitoylation decreased in a statistically significant manner during a migration time course (Figure 5A,B). This was accompanied by a notable increase in CD44 co-precipitation with its pro-migratory binding partner ezrin at 1 to 2 hours (Figure 5C). Similarly, CD44/ezrin co-precipitation was increased in migrating cells expressing a CD44 double-site palmitoylation mutant (Figure 5D).

Having shown a direct relationship between CD44 palmitoylation status and cell migration, we translated this into a more patient-relevant context by evaluating CD44 palmitoylation and raft affiliation status in representative primary cultures isolated from non-invasive versus invasive human breast tumours. Cultures derived from non-invasive ductal carcinoma *in situ* tumours formed organised colonies of luminal-like epithelial cells surrounded by flattened, myoepithelial-like cells (Figure 5E, phase contrast). Cultures derived from invasive ductal carcinoma tumours contained predominantly flattened protrusive cells. CD44 co-localised extensively with Flotillin-1 in the cell membranes of ductal carcinoma *in situ* cultures (Figure 5E), but there was little spatial overlap of these proteins in invasive ductal carcinoma cultures. Accordingly, lower levels of palmitoylated CD44 were detected in whole cell extracts from six representative invasive ductal carcinoma cultures relative to two representative nontumour cultures (Figure 5F). Collectively, these results suggest a novel clinical relevance for the regulation of palmitoylation and lipid raft affiliation of CD44 in breast cancer cells.

## Discussion

Cell migration is an early molecular event in breast cancer metastasis, and identifying tumour markers that correlate with metastatic potential is a focus of numerous studies. CD44 has the ability to interact with numerous components of the extracellular matrix, thereby facilitating cell migration and local invasion. CD44 has been described previously as both a tumour suppressor [9] and an oncoprotein [7] in various cancers, which highlights a complex and probably tissue-specific role in oncogenesis. Despite expressional correlations of CD44 [24,25] or its splice variants [7] with progression of aggressive breast cancers, specific mechanisms of CD44-dependent cell migration remain controversial. CD44 resides in lipid rafts [6,26,27], organisation centres for molecules that play key roles in cell migration and whose altered functional behaviour has been implicated in diseases including breast cancer [12]. We have shown that CD44 localisation in rafts limits associations with its cytoskeletal linker binding partner ezrin [18]. We therefore hypothesised that translocation of CD44 outside rafts to bind to migratory partners (such as ezrin) is a novel regulatory mechanism controlling CD44-dependent cell migration. In the present study we dissected the relationship between CD44 raft affiliation, ezrin association and migratory potential in breast cancer cells.

Although hyaluronan has previously been used to stimulate CD44-dependent migration [16,18,28], in this study we concentrated on ligand-independent breast cancer cell migration induced via scratch-wounding. Having previously shown reduced CD44 raft affiliation during migration of invasive MDA-MB-231 and Hs578T breast cells [18], here we found that, in the normal-like breast epithelial cell line MCF-10a, CD44 affiliation with lipid rafts was in fact *increased* under migratory conditions. This may suggest that lipid rafts sequester CD44 to limit the migration of normal-like cells. Accordingly, the importance of lipid raft domains in maintaining front–rear polarity during breast cancer cell migration has been highlighted [29]. Also noteworthy is the exclusive lipid raft recovery of what putatively are heavier CD44 isoforms in MCF-10a cells. MCF-10a cells express predominantly the CD44 standard isoform, but v8-10, v3-10 and v2-10 mRNAs have also been detected [7]. Since our focus was on the CD44 standard isoform, we did not characterise which variants might be present in our model. However, since changes in CD44 expression during cell migration were observed only in lipid raft fractions of this weakly motile cell line, it is tempting to speculate that nonmalignant polarised cells sequester proteins such as CD44 inside lipid rafts in order to limit cell migration. In support of this hypothesis, heavier CD44 isoforms have been reported in lipid rafts along with CD44s and annexin II in the nontumorigenic breast epithelial cell line, EpH4 [30]. In view of intriguing evidence that various CD44 isoforms may associate with different histological parameters of breast cancer [7], it will be interesting to probe the relative raft/nonraft expression of these variants in response to cell migration in future studies.

In contrast to MCF-10a cells, CD44 lipid raft affiliation in the metastatic cell line MDA-MB-231 was almost halved during cell migration. Additionally, we observed a lower content of CD44 in nonraft fractions of Hs578T cells relative to that in its more invasive isogenic derivative Hs578Ts(i)_8_ (data not shown). These observations support our hypothesis of a direct relationship between increased extra-raft localisation of CD44 and a more invasive/migratory breast cancer cell phenotype. To our knowledge, these are the first reports of a link between CD44 raft localisation and breast cancer cell migration. However, localisation of CD44 outside lipid rafts in human glioblastoma cells has been shown to induce metalloproteinase-mediated CD44 shedding and tumour cell migration [31], which was further confirmed after membrane cholesterol modulation with methyl-β-cyclodextrin, filipin and simvastatin [32]. In contrast, raft affiliation of CD44 has been demonstrated to recruit Src and integrins in gastric and colorectal cancers, promoting cell survival and increasing endothelial adhesion [33]. Previous reports from the Isacke group have suggested that alterations in cell motility do not alter CD44 associations with detergent-insoluble cellular pools [11]. However, under their experimental conditions, random, nondirectional migration/spreading was induced in subconfluent cells in response to scatter factor, whereas our conditions induced directional cell migration in fully confluent cells via ligand-independent wound closure methodologies. Any perceived discrepancies between the studies are thus likely to be context dependent, but we speculate that CD44 redistribution outside rafts is associated with directional motility while perhaps being less important for random motility. The potential implications of this for cell migration out of an *in vivo* tumour are significant, but are not well understood. It is intriguing to speculate that our directional cell migration model might recapitulate the phenomenon of Indian file collective cell migration out of certain primary breast tumours; while nondirectional models of cell migration might mimic that of single-cell amoeboid-like migration out of primary tumours. That said, our findings *vis-à-vis* the association between extra-raft CD44 and invasion/migration are broadly supported by data in primary cultures from breast cancer patients. This represents an important pathophysiological context that cannot be replicated in immortalised cell cultures. Taken together, our overall data in breast cells suggest that CD44 localisation outside lipid rafts is associated with a pro-migratory state; furthermore, a range of CD44 binding partners was only ever recovered from nonraft domains. Given the reported involvement of ezrin [34] and ankyrin [35] in cancer progression and of merlin in tumour suppression [17], we speculate that the spatial availability of CD44 to interact with specific cytosolic partners may differentially regulate cancer cell migration.

Dual acylation of two cysteine residues (Cys286 and Cys295) in the CD44 transmembrane region has been shown to be required for its raft association in fibroblasts and embryonic kidney cells [36]. We thus used transient overexpression of palmitoylation-impaired mutants as a tool to decrease CD44 affiliation with lipid rafts. Triton X-100 insolubility assays were used as a surrogate for lipid raft extractions, since detergent-insoluble fractions have been shown to be enriched in lipid rafts [32,37]. Overexpression of CD44 palmitoylation-impaired mutants reduced the affiliation of CD44 with rafts, but CD44WT-overexpressing cells also presented decreased CD44 raft affiliation. This may reflect enhanced CD44 trafficking, since we observed a coincident increase in vesicular/cytoplasmic staining of an early endosomal marker in CD44WT cells (data not shown). It is also noteworthy that raft affiliation of *total* CD44, encompassing the endogenous pool in addition to the exogenously expressed pool, appeared to be reduced upon expression of CD44WT or CD44 mutants. Although the mechanism whereby endogenous CD44 might accordingly reduce its affiliation with rafts is unknown, one speculation is that it reflects hetero-dimerisation between endogenous and overexpressed CD44 and their consequent co-trafficking in/out of rafts. However, Neame and colleagues have previously shown that transfected CD44 does not associate with endogenous CD44 [11]. Nevertheless, CD44 has been shown to homo-dimerise on activated leukocytes via Cys286 [38] and to hetero-dimerise with growth factor receptors [39]. Since point mutation of Cys286 in our experimental system might reduce potential dimerisation events, a possible alternative is that the overexpression of wild-type or mutant CD44 simply exerts a negative tone on the overall expression of endogenous CD44. While this could be tested by incorporating specific tags onto endogenous CD44 versus exogenous CD44, the fact that the tags might themselves influence raft/nonraft partitioning [40] complicates the clarity of both question and answer. Nonetheless, despite only using a transient transfection approach featuring very modest levels of CD44 overexpression, the important point is that distinct functional differences were observed in MDA-MB-231 cells as a direct consequence of reduced CD44 raft affiliation. Specifically, migration was higher in cells expressing CD44 palmitoylation mutants relative to controls or CD44WT-expressing cells. It is also noteworthy that higher cell migration in CD44WT versus control cells was paralleled by lower levels of palmitoylated CD44 in the former. The published links between CD44WT overexpression and cell migration/invasion in breast cancer cell lines [41] and patient samples [14] may thus also reflect a proportional decrease in CD44 palmitoylation status.

Our data have also revealed that forcing CD44 outside lipid rafts with palmitoylation mutants was sufficient to enhance cell migration and to induce an appearance reminiscent of EMT in histologically normal MCF-10a breast cells. Cells overexpressing CD44WT also acquired some morphological features of EMT (in terms of a more scattered/migratory phenotype), but over a longer time course. Although we did not quantitate cell scattering or extensively characterise the relative phenotypes of mutants versus controls, our morphological observations are consistent with studies showing decreased epithelial markers and increased mesenchymal markers upon CD44 activation [42], and links between CD44, EMT and breast cancer progression [42]. That our observed effects related specifically to CD44 mutant expression was confirmed upon termination of selection. Furthermore, this served as an isogenic control for the inevitable differences in CD44 expression that occur between separate cell lines. Such expressional differences were evident in HMEC, MCF7 and BT-474 breast cells (all of which were tested in addition to the ones discussed in this report), and which accordingly complicated the generation of a single paradigm relating CD44 raft/nonraft affiliation to migration across all cell lines. Interestingly, EMT in MCF-10a cells has been linked to a switch between a high molecular weight CD44 isoform and the standard isoform [42]. It is unknown where this transition should take place; however, we noted reduced expression of high molecular weight CD44 in palmitoylation mutant cells. Furthermore, CD44 localisation in lipid rafts and levels of palmitoylation in nontumour versus invasive breast cancer primary cultures supports the conclusions we have made in normal-like versus highly invasive breast cancer cell lines. Taken together, this might suggest that, in the early stages of cancer progression, the standard form of CD44 becomes prevalent and localises outside lipid rafts in order to bind its oncogenic binding partners and stimulate cell migration.

Having shown that CD44 palmitoylation inversely regulates cell migration, we sought to examine whether migration itself might alter CD44 palmitoylation. We present novel evidence that CD44 palmitoylation is temporally reduced during breast cancer cell migration. Although the mechanisms for this reduction remain elusive, it is possible that actin rearrangements during migration facilitate altered spatial access of palmitoylating or de-palmitoylating enzymes to targets such as CD44. More importantly, reductions in CD44 palmitoylation were paralleled by enhanced co-association of CD44 with the linker protein ezrin, providing a direct mechanism for cytoskeletal engagement in order to drive cell migration. The potential clinical significance of this observation was illustrated by the fact that CD44 palmitoylation levels were low in primary cultures from human invasive ductal carcinomas in comparison with nontumour tissue; and that co-localisation of CD44 with the lipid raft marker Flotillin-1 was also low in invasive specimens. We are therefore confident that changes in CD44 raft affiliation via palmitoylation alterations is not an artefact of *in vitro* manipulations, but rather a physiological phenomenon that merits further investigation.

## Conclusion

**Figure 6:**
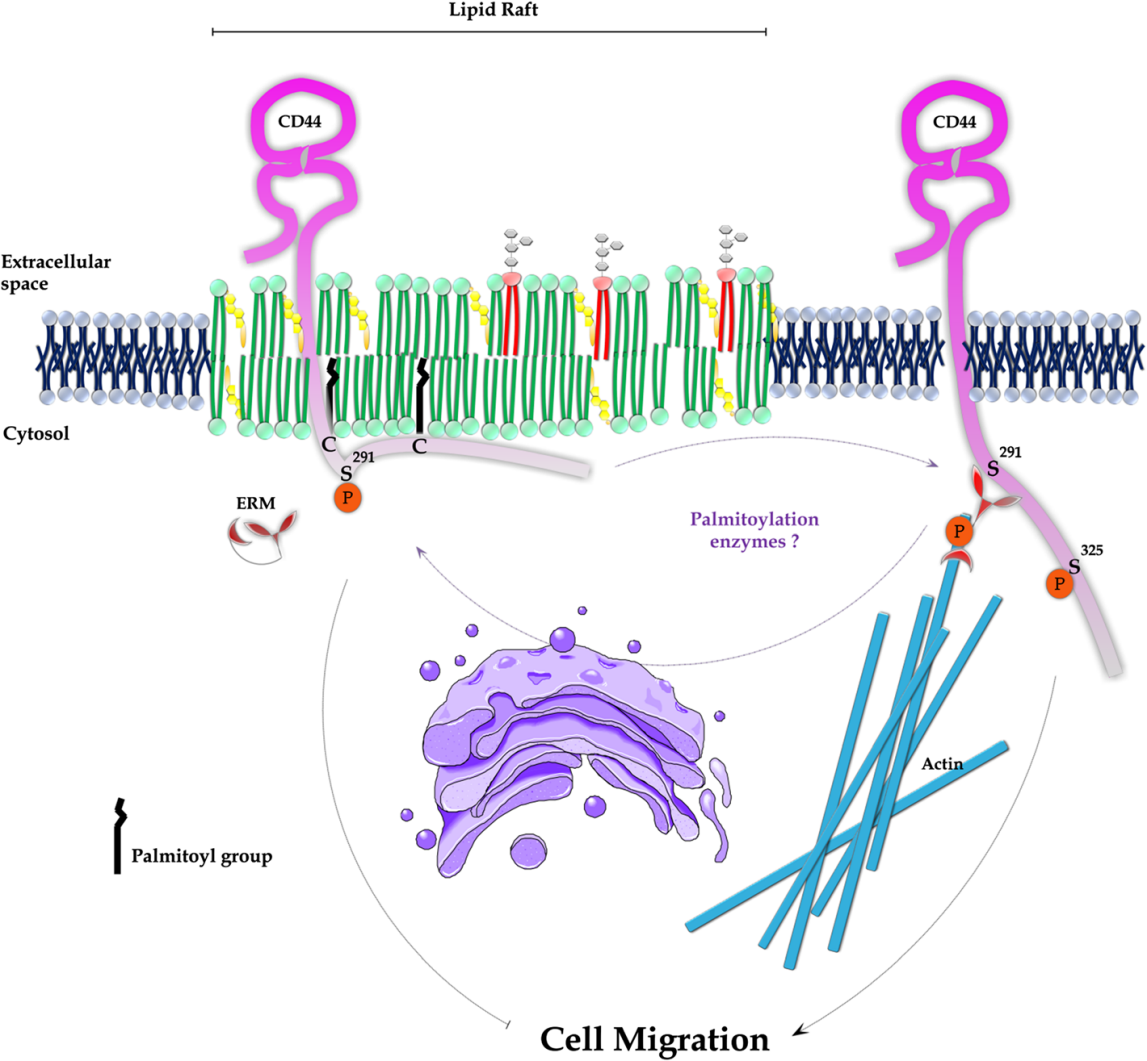
**Schematic representation of the proposed model of regulation of breast cancer cell migration via CD44 localisation in lipid rafts.** When CD44 is affiliated with lipid rafts via palmitoylation of its cysteine residues, it is sequestered from binding its cytoplasmic binding partners and thus migration is restrained. However, when CD44 translocates outside lipid rafts in its de-palmitoylated state, its cytoplasmic tail is free to bind its cytoskeletal partners, subsequently facilitating cell migration. ERM, Ezrin/Radixin/Moesin.

Our findings are consistent with a novel model in which CD44 palmitoylation facilitates its sequestration within lipid rafts, restricting its availability to bind to pro-migratory binding partners such as ezrin (Figure 6) and thereby restraining tumourigenic behaviour. We submit that further studies into mechanisms underlying the regulation of CD44 palmitoylation and lipid raft containment may merit evaluation as a novel target to reduce breast cancer metastatic spread.

## Abbreviations

BMCC: 1-biotinamido-4-(4′-(maleimidomethyl)cyclohexanecarboxamido)butane; EMT: epithelial-to-mesenchymal transition; HRP: horseradish peroxidase; TfR: transferrin receptor.

## Competing interests

The authors declare that they have no competing interests.

## Authors’ contributions

ISB participated in the study design, performed most of the experimental work, analysed the data, drafted the manuscript and critically revised it. EAMcS performed part of the experimental work and participated in design, data interpretation and drafting of the revised manuscript. SD performed the primary culture isolations and contributed to study design, data interpretation and critical analysis. ADKH participated in interpretation of the data and revised it critically for intellectual content. AMH conceived and designed the study, interpreted the data, co-drafted and critically revised the manuscript. All authors read and approved the final manuscript.

## Supplementary Material

Additional file 1 **is Figure S1 showing transfected CD44 DNA expression was confirmed in MDA-MB-231 cells. (A)** DNA from MDA-MB-231 cells transfected with CD44 mutants C286A and C286S,295A (SA) in a pOTB7 vector and selected with chloramphenicol was extracted, polymerase chain reaction (PCR)-amplified using primers specific to C286A (lane 2) and SA (lane 3) and 100 ng purified product separated on a 1% agarose gel. The probable presence of mutant CD44 in MDA-MB-231 cells (in the appropriate size ranges) was confirmed, while the negative control lane (no template DNA) remained blank. **(B)** Successful expression of both the C286A and SA mutants in transfected MDA-MB-231 cells was confirmed by sequencing of the purified PCR products (50 ng). Click here for file

Additional file 2 **is Figure S2 showing overexpression of CD44 or its palmitoylation-impaired mutants induces an EMT-like state.** MCF-10a cells were transfected for 48 hours with CD44WT, single-site (C286A) or double-site (AA) palmitoylation-impaired mutant constructs. Immunoblotting for the epithelial marker EpCAM and the mesenchymal marker vimentin suggested an EMT-like switch in cells overexpressing CD44WT or its palmitoylation mutants. Click here for file

Additional file 3 **is Figure S3 showing biochemical and functional phenotypes in cells overexpressing CD44 palmitoylation-impaired mutants are reversible.** Following 48-hour expression of CD44WT or palmitoylation-impaired single (C268A, C286S) or double (SA, AA) mutants in MDA-MB-231 and MCF-10a cells, the cells were subcultured and grown without selection reagent for a further 48 hours. **(A)** After termination of CD44WT or mutant selection in MCF-10a cells, CD44 recovery from Triton X-100-insoluble fractions was restored to match that of control cells. **(B)** Lack of statistically-significant differences *(ns, not significant, Student’s t test)* between the raft affiliation ratio of CD44 in control MCF-10a cells, versus those in which mutant selection had been terminated, confirmed restoration of a normal biochemical phenotype. **(C)** Scratch-wound assays confirmed that cell migration returned to control levels in MCF-10A cells following termination of expression of CD44WT or palmitoylation-impaired mutants *(two-way analysis of variance (ANOVA))*. **(D)** After termination of CD44WT or mutant selection in MDA-MB-231 cells, CD44 recovery from Triton X-100-insoluble fractions was restored to match that of control cells *(ns, not significant, Student’s t test)*. **(E)** Scratch-wound assays confirmed that cell migration returned to control levels in MDA-MB-231 cells following termination of expression of CD44WT or palmitoylation-impaired mutants *(two-way ANOVA). Error bars, standard error of the mean; n = 3 experiments.* Click here for file
