# Supplementary figures and images for: A novel mechanism of regulating breast cancer cell migration via palmitoylation-dependent alterations in the lipid raft affiliation of CD44
Source: Breast Cancer Res. 2014 Feb 10;16(1):R19. doi: 10.1186/bcr3614 (PMC3978828; doi:10.1186/bcr3614)

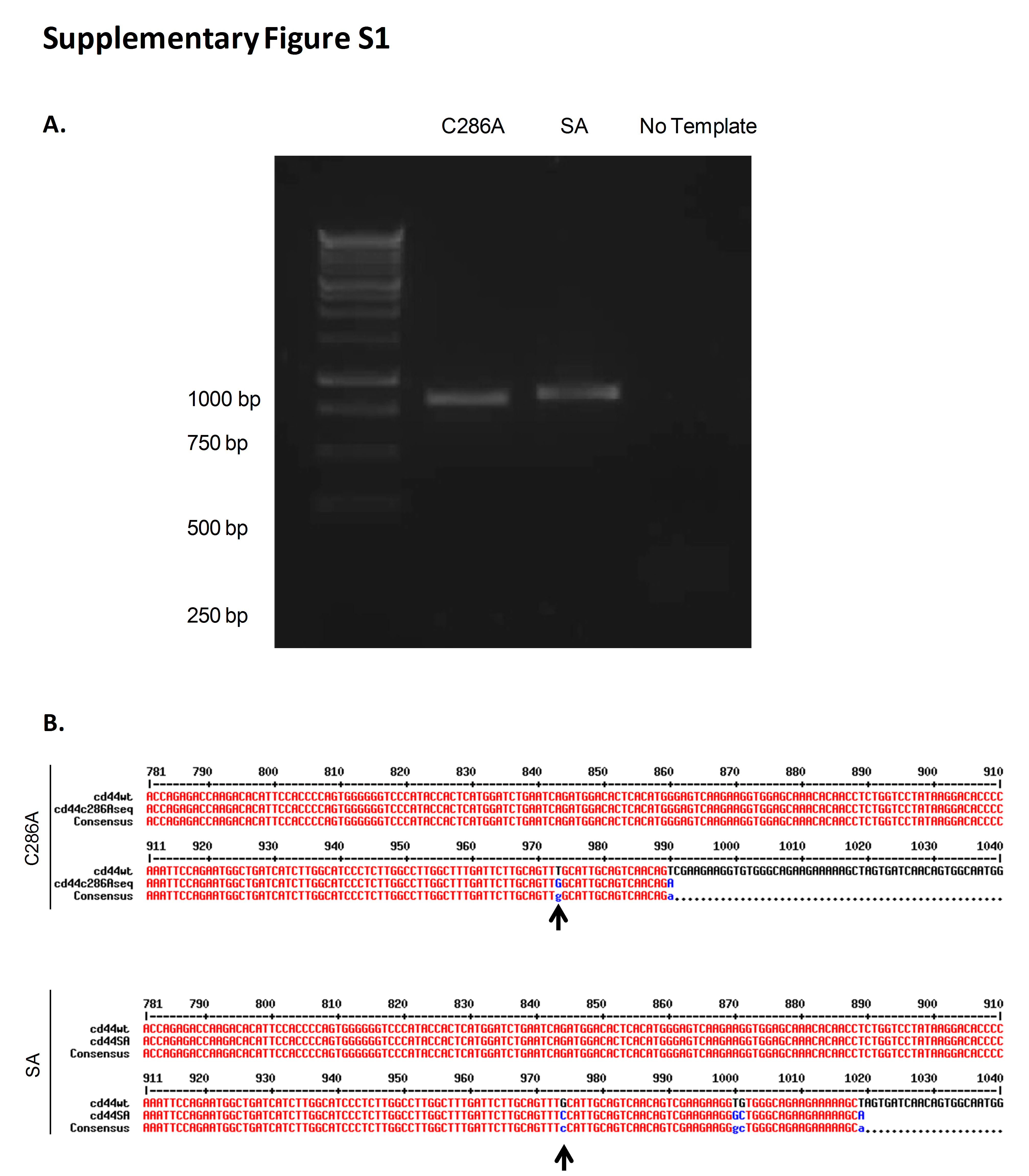

Supplement: Additional file 1 — is Figure S1 showing transfected CD44 DNA expression was confirmed in MDA-MB-231 cells. (A) DNA from MDA-MB-231 cells transfected with CD44 mutants C286A and C286S,295A (SA) in a pOTB7 vector and selected with chloramphenicol was extracted, polymerase chain reaction (PCR)-amplified using primers specific to C286A (lane 2) and SA (lane 3) and 100 ng purified product separated on a 1% agarose gel. The probable presence of mutant CD44 in MDA-MB-231 cells (in the appropriate size ranges) was confirmed, while the negative control lane (no template DNA) remained blank. (B) Successful expression of both the C286A and SA mutants in transfected MDA-MB-231 cells was confirmed by sequencing of the purified PCR products (50 ng). [file bcr3614-S1.tiff]

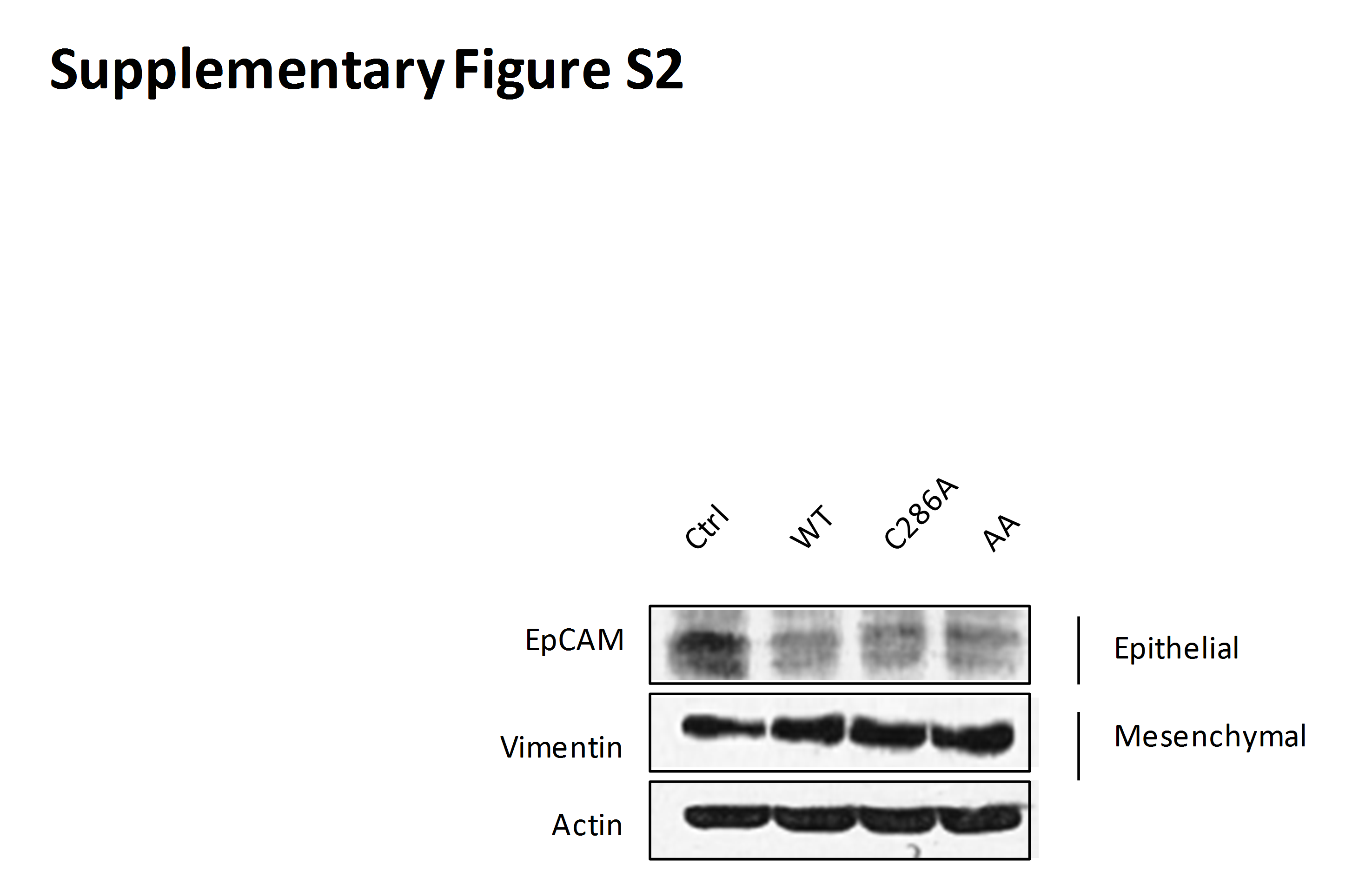

Supplement: Additional file 2 — is Figure S2 showing overexpression of CD44 or its palmitoylation-impaired mutants induces an EMT-like state. MCF-10a cells were transfected for 48 hours with CD44WT, single-site (C286A) or double-site (AA) palmitoylation-impaired mutant constructs. Immunoblotting for the epithelial marker EpCAM and the mesenchymal marker vimentin suggested an EMT-like switch in cells overexpressing CD44WT or its palmitoylation mutants. [file bcr3614-S2.tiff]

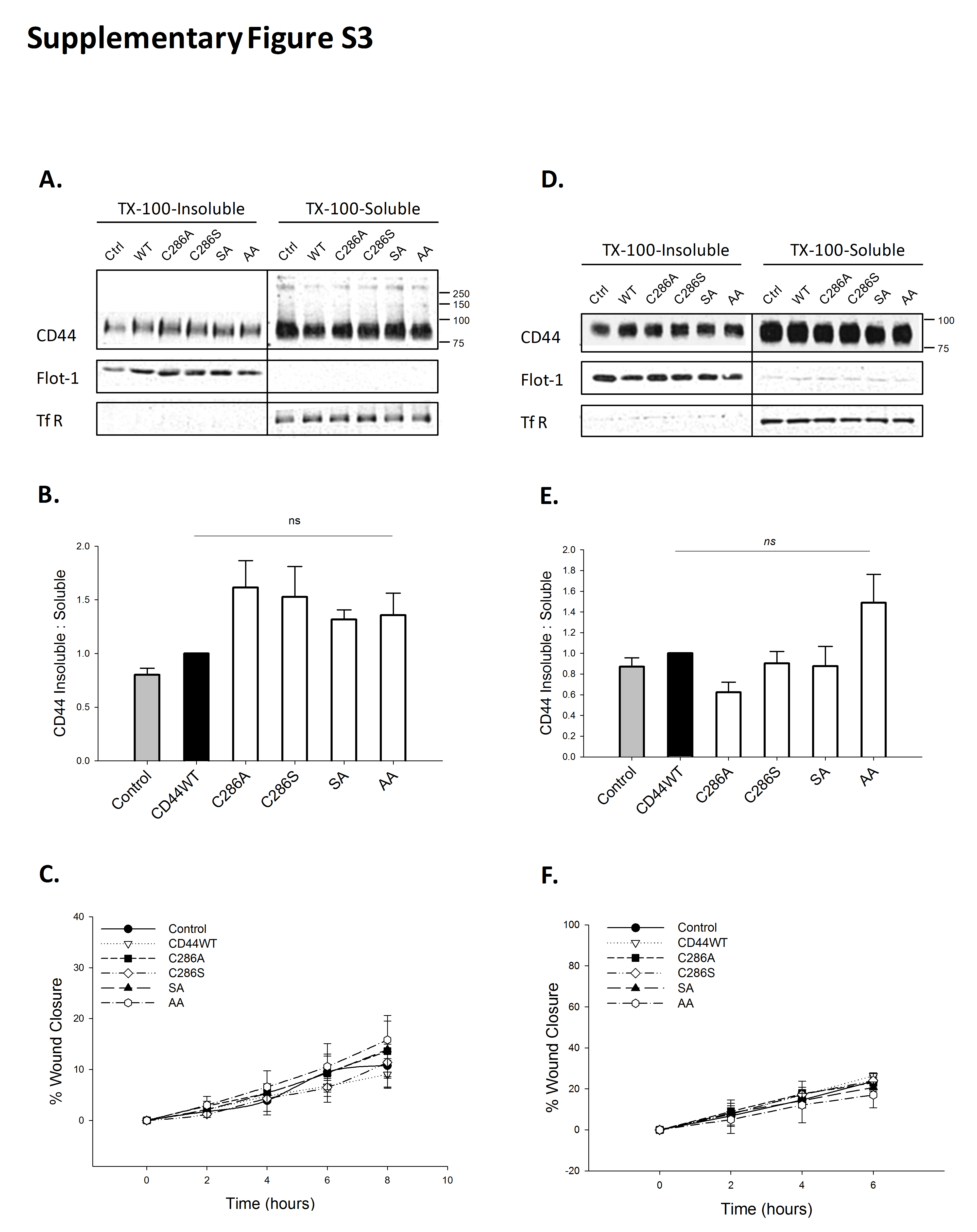

Supplement: Additional file 3 — is Figure S3 showing biochemical and functional phenotypes in cells overexpressing CD44 palmitoylation-impaired mutants are reversible. Following 48-hour expression of CD44WT or palmitoylation-impaired single (C268A, C286S) or double (SA, AA) mutants in MDA-MB-231 and MCF-10a cells, the cells were subcultured and grown without selection reagent for a further 48 hours. (A) After termination of CD44WT or mutant selection in MCF-10a cells, CD44 recovery from Triton X-100-insoluble fractions was restored to match that of control cells. (B) Lack of statistically-significant differences (ns, not significant, Student’s t test) between the raft affiliation ratio of CD44 in control MCF-10a cells, versus those in which mutant selection had been terminated, confirmed restoration of a normal biochemical phenotype. (C) Scratch-wound assays confirmed that cell migration returned to control levels in MCF-10A cells following termination of expression of CD44WT or palmitoylation-impaired mutants (two-way analysis of variance (ANOVA)). (D) After termination of CD44WT or mutant selection in MDA-MB-231 cells, CD44 recovery from Triton X-100-insoluble fractions was restored to match that of control cells (ns, not significant, Student’s t test). (E) Scratch-wound assays confirmed that cell migration returned to control levels in MDA-MB-231 cells following termination of expression of CD44WT or palmitoylation-impaired mutants (two-way ANOVA). Error bars, standard error of the mean; n = 3 experiments. [file bcr3614-S3.tiff]
